# TissUnet: Improved extracranial tissue and cranium segmentation for children through adulthood

**DOI:** 10.1162/IMAG.a.1067

**Published:** 2026-01-05

**Authors:** Markiian Mandzak, Elvira Yang, Anna Zapaishchykova, Yu-Hui Chen, Lucas Heilbroner, John Zielke, Divyanshu Tak, Reza Mojahed-Yazdi, Francesca Romana Mussa, Zezhong Ye, Sridhar Vajapeyam, Viviana Benitez, Ralph Salloum, Susan N. Chi, Houman Sotoudeh, Jakob Seidlitz, Sabine Mueller, Hugo J.W.L. Aerts, Tina Y. Poussaint, Benjamin H. Kann

**Affiliations:** Artificial Intelligence in Medicine (AIM) Program, Mass General Brigham, Harvard Medical School, Boston, MA, United States; Department of Radiation Oncology, Dana-Farber Cancer Institute and Brigham and Women’s Hospital, Harvard Medical School, Boston, MA, United States; Ukrainian Catholic University, Lviv, Ukraine; Ludwig Maximilian University of Munich, Munich, Germany; Radiology and Nuclear Medicine, CARIM & GROW, Maastricht University, Maastricht, the Netherlands; Department of Data Science, Dana-Farber Cancer Institute, Boston, MA, United States; George Washington School of Medicine and Health Sciences, Washington, DC, United States; Boston Children’s Hospital, Boston, MA, United States; Department of Pediatric Oncology at Dana-Farber Cancer Institute, Boston, MA, United States; UT Southwestern Medical Center, Dallas, TX, United States; Lifespan Brain Institute, The Children’s Hospital of Philadelphia and Penn Medicine, Philadelphia, PA, United States; Department of Psychiatry, University of Pennsylvania, Philadelphia, PA, United States; Department of Child and Adolescent Psychiatry and Behavioral Science, The Children’s Hospital of Philadelphia, Philadelphia, PA, United States; Institute for Translational Medicine and Therapeutics, University of Pennsylvania, Philadelphia, PA, United States; Department of Neurology, Neurosurgery and Pediatrics, University of California, San Francisco, CA, United States

**Keywords:** whole-head segmentation, MRI, deep learning, artificial intelligence, pediatric brain tumor

## Abstract

Extracranial tissues visible on brain magnetic resonance imaging (MRI) may hold significant value for characterizing health conditions and clinical decision-making, yet they are rarely quantified. Current tools have not been widely validated, particularly in settings of developing brains or underlying pathology. We present TissUnet, a deep learning model that segments skull bone, subcutaneous fat, and muscle from routine three-dimensional T1-weighted MRI, with or without contrast enhancement. The model was trained on 155 paired MRI–computed tomography (CT) scans and validated across nine datasets covering a wide age range and including individuals with brain tumors. In comparison to AI-CT-derived labels from 37 MRI–CT pairs, TissUnet achieved a median Dice coefficient of 0.79 [IQR: 0.77–0.81] in a healthy adult cohort. In a second validation using expert manual annotations, median Dice was 0.83 [IQR: 0.83–0.84] in healthy individuals and 0.81 [IQR: 0.78–0.83] in tumor cases, outperforming a previous state-of-the-art method. Acceptability testing resulted in an 89% acceptance rate after adjudication by a tie-breaker (N = 108 MRIs), and TissUnet demonstrated excellent performance in the blinded comparative review (N = 45 MRIs), including both healthy and tumor cases in pediatric populations. TissUnet enables fast, accurate, and reproducible segmentation of extracranial tissues, supporting large-scale studies on craniofacial morphology, treatment effects, and cardiometabolic risk using standard brain T1w MRI.

## 1 Introduction

Magnetic Resonance Imaging (MRI) is a widely used and standard imaging modality for visualizing brain anatomy, playing a central role in clinical care and neuroscience research. In particular, children and adults with neurologic conditions, such as brain tumors, multiple sclerosis, or dementia, undergo frequent MRI scans throughout diagnosis, treatment, and survivorship. In these instances, much focus is given to intracranial pathology, both qualitatively and quantitatively, motivating the development of many deep learning-based tools for intracranial brain and pathology segmentation (Stolte et al., 2024; Tierney et al., 2025).

Emerging evidence suggests that extracranial features may carry clinically meaningful, opportunistic information, including markers of treatment toxicity, physiologic reserve, and long-term outcomes (Cho et al., 2022; Hsieh et al., 2019; Zapaishchykova, Liu, et al., 2023; Zhang et al., 2023). Quantification and longitudinal tracking of these tissues would be clinically valuable, yet are impractical and challenging to do manually. Deep learning-based segmentation is a promising strategy for practical, accurate extracranial tissue segmentation, but there are no publicly available tools that enable comprehensive, three-dimensional segmentation of extracranial structures in standard brain MRIs, particularly for pediatric populations or those with brain pathology. This gap is especially relevant given the particular importance of sarcopenia in pediatric brain tumor (PBT) survivors, which leads to devastating physiologic frailty in up to 30% of survivors (Joffe et al., 2019) and is associated with reduced neurocognitive function, quality of life, and survival (Mager et al., 2023; Schulte et al., 2010). While physiologic frailty is well-characterized in adults, it remains poorly defined in children due to age- and puberty-related variability. Current clinical assessment relies on indirect metrics such as body mass index (BMI), which lacks specificity and correlates poorly with outcomes across pediatric populations (Marković-Jovanović et al., 2015).

Despite the growing interest in MRI-based body composition as a prognostic marker in these patients, few efforts have addressed the practical barriers to extracranial segmentation at scale. A major challenge is the ground-truth labels as manual annotation of extracranial structures is time-intensive and technically demanding (Galbusera & Cina, 2024). Although recent tools such as GRACE (Stolte et al., 2024) have demonstrated the potential of automated segmentation, no existing automated segmentation tools have been validated across the entire human lifespan, in particular pediatric populations and in the presence of pathology. This represents a significant gap, as accurate segmentation must account for the anatomical variability that occurs across different life stages. Further compounding these issues is the inconsistent application of defacing algorithms, which are commonly used in publicly shared MRI datasets to protect patient identity (Familiar et al., 2024). These methods vary widely in how much facial and extracranial tissue is removed, undermining model generalizability and limiting downstream biomarker discovery.

To address these challenges, we present a contrast-invariant T1w MRI deep learning (DL) framework, *TissUnet,* for automated segmentation of major extracranial tissues: bone (skull), subcutaneous fat, and muscle. In addition to volumetric analysis, our pipeline supports downstream anthropometric measurements of skull thickness derived from anatomical landmarks. We compare TissUnet against current state-of-the-art (SoTa) methods and multiple skull thickness estimation methods. To enable consistent analysis across heterogeneous, publicly available MRIs, we introduce a brain mask–guided region-of-interest (ROI) cropping strategy that isolates relevant extracranial structures while mitigating the effects of defacing and scanner variation. We further demonstrate its potential clinical application by modeling associations between tissue volume, body composition, and lipid profiles in adolescents.

## 2 Materials and Methods

The research was deemed not to require IRB oversight as it consists of secondary analysis of de-identified primary datasets.

**Fig. 1.**
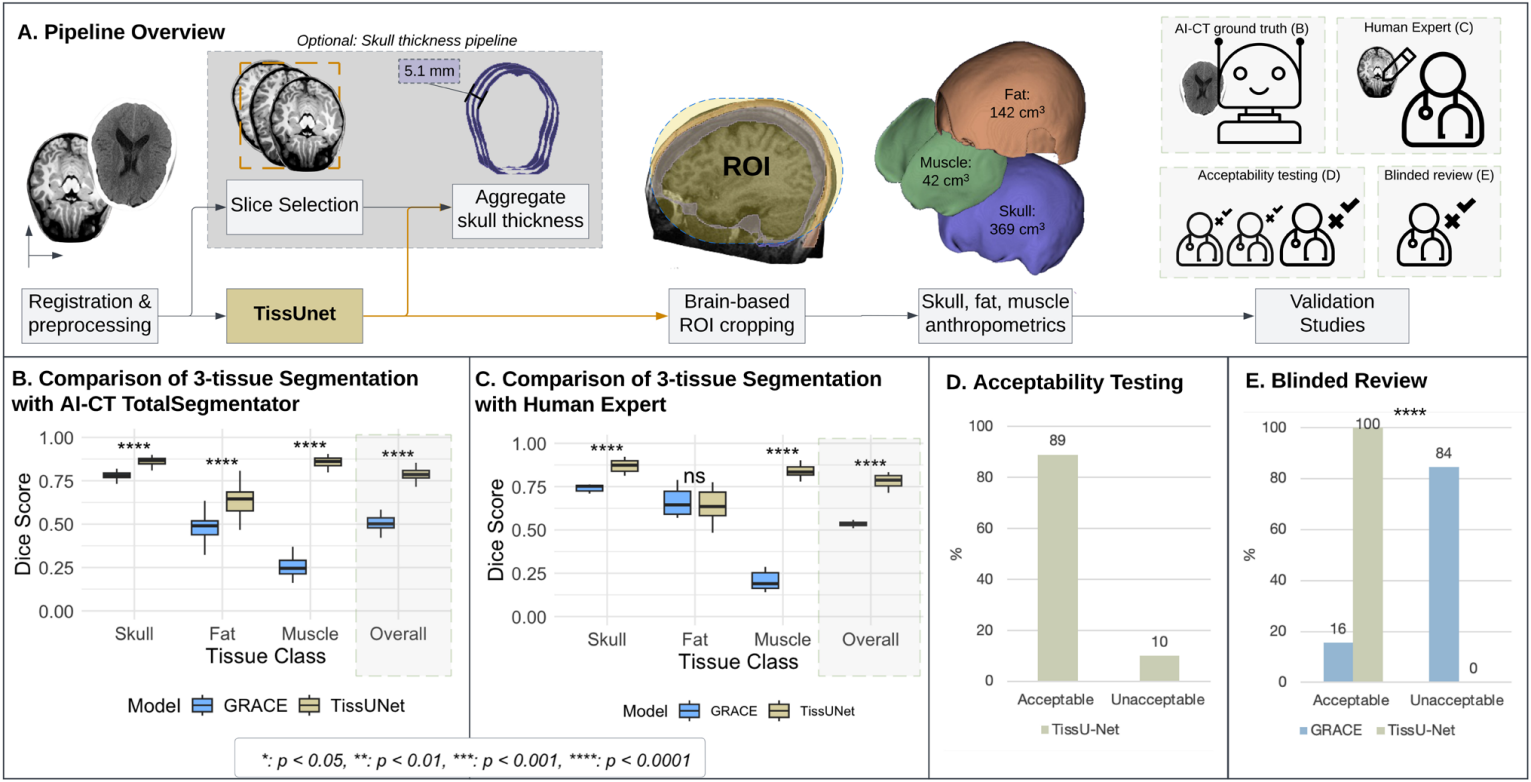
(A) TissUnet pipeline overview. Step 1: The MRI T1w images are registered to the corresponding age-based NIHPD template. Step 2: TissUnet predicts 4 classes: brain, skull, subcutaneous fat, and muscle. Step 3: Using a brain mask, a universal ROI for calculating tissue volumes is created. Step 4: Skull, subcutaneous fat, and muscle anthropometric measurements are calculated on the cropped ROI. Optional: To estimate skull thickness, we used the DenseNet model to pick the top orbital roof slices and aggregate skull thickness measurements estimated from 95% measured tangents from 100 points at each 16 × 1 mm 2D axial slice. For validation studies, we created four different experiments: AI-CT ground truth (B), Human Expert annotations (C), Acceptability testing (D), and Blinded Review (E). (B) Boxplot of Dice of two DL methods performance compared to AI segmentations generated over CT images on a three-class tissue segmentation task. TissUnet (yellow), GRACE (blue) for fat, bone, and muscle, and overall, between three classes on MRI T1w (N = 37, healthy CERMEP dataset). See Table 1 for Dice and HD95 medians and IQR. Pairwise significance was tested with the Mann–Whitney U test with FDR correction for multiple testing. IQR = interquartile range. (C) Boxplot of Dice of two DL methods performance compared to human expert segmentations on a three-class tissue segmentation task. TissUnet (yellow), GRACE (blue) for fat, bone, muscle, and overall between three classes on MRI T1w (N = 10, 5 healthy and 5 brain tumor cases). The Dice score ranges from a minimum of 0 (worst score) to a maximum of 1 (best score). Box plots representing the interquartile range for each method per tissue. See Table 2 for Dice and HD95 by health status. Pairwise significance was tested with the Mann–Whitney U test with FDR correction for multiple testing. Bar plots for acceptability testing (N* = *108 cases, 54 healthy and 54 brain tumor cases). See Table 3 for intra-rater agreement scores by diagnosis and tissue class (skull, fat, muscle). (E) Bar plot for blinded review (N = 54 MRI, 34 healthy and 12 with brain tumor). All cases (N = 45, 100%) were rated acceptable using TissUnet, with none deemed unacceptable. In GRACE, 7 cases (16%) were rated acceptable and 38 cases (84%) were rated unacceptable. p-values measured using Chi-Squared.

**Fig. 2.**
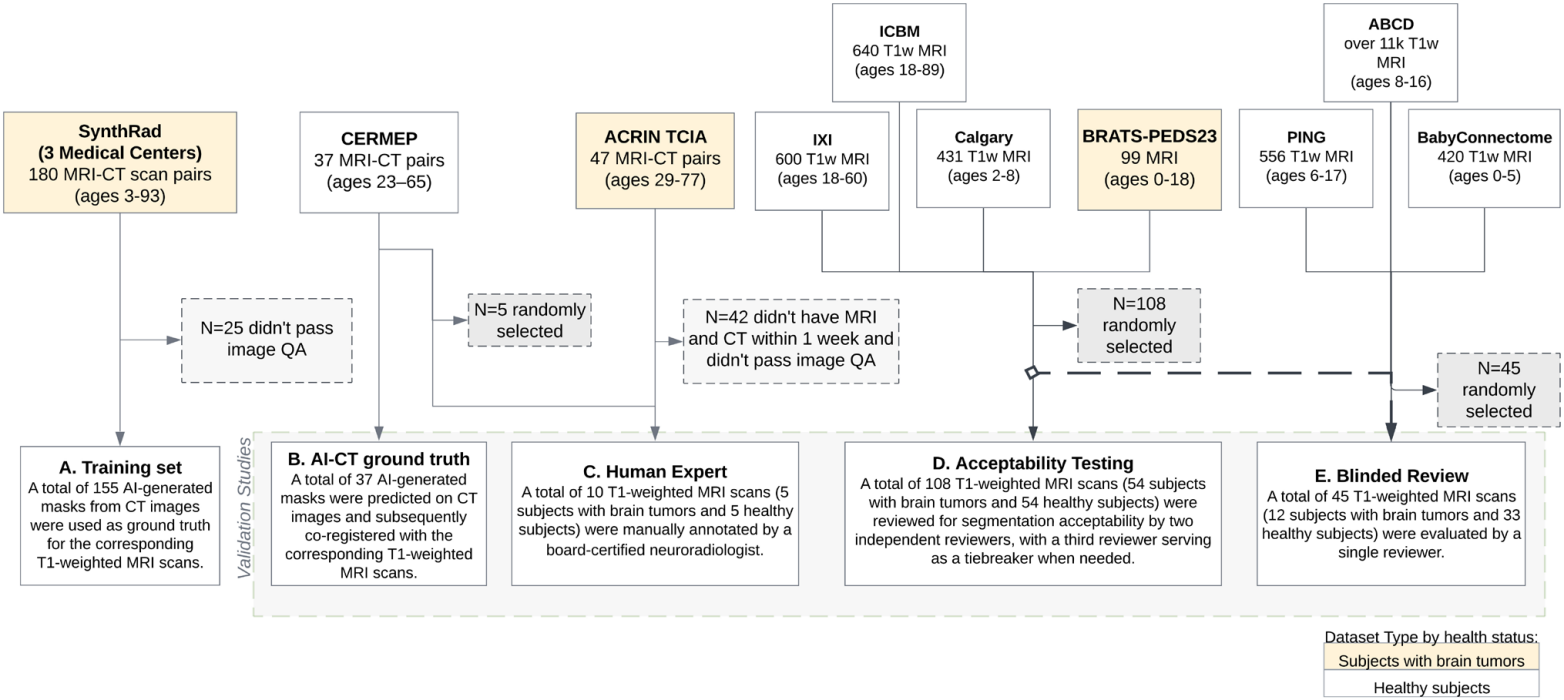
Cohort selection by dataset origin and health status for TissUnet training and testing settings. In yellow labeled datasets with subjects with brain tumors, in white datasets with healthy subjects.

TissUnet is a multitask, neural network trained to segment three extracranial tissues—skull, fat, and muscle via 3-dimensional, T1-weighted (T1w) brain MRI (Fig. 1A). Additionally, the model outputs 3D volumetrics of each tissue as well as an estimate of skull thickness. The model was developed to perform accurately across the human lifespan and in the presence of intracranial pathology. To address variability introduced by defacing algorithms and scanner differences, the pipeline employs a novel brain mask–based region-of-interest (ROI) cropping pipeline. To test the proposed method’s robustness toward the registration deviations from the template, we rotate MRI T1w 5 degrees anterior and 5 degrees posterior and compare fat, muscle, and bone volumetric differences. Ten publicly available datasets were used for this study (Fig. 2; Supplementary Methods 1) under data use agreements where necessary.

### 2.1 Training dataset and label generation

TissUnet, based on the nnU-Net v2 framework (Wasserthal et al., 2023), was trained using a multi-center dataset SynthRAD2023, comprising 180 patients with brain tumors with co-registered CT-MRI T1w pre- and post-contrast imaging pairs (64% (N = 115) male, mean age 65, range 3−93 years (Thummerer et al., 2023) (Fig. 2A). Since muscle, fat, and bone are readily visible on CT, and due to the time and cost associated with generating de novo ground truth segmentations on MRI, we used segmentations generated by a previously validated CT-based AI algorithm, TotalSegmentator (Wasserthal et al., 2023), as initial ground truth labels that were then propagated to the co-registered T1w MRI.

To generate training pseudolabels for extracranial tissues, we applied the “head muscles,” “oculomotor muscles,” and “tissue types” models from TotalSegmentator (Wasserthal et al., 2023) to CT scans from paired MRI–CT datasets. The raw labels were then merged into three target classes for training: (1) muscle: temporalis left, temporalis right; (2) bone: skull; and (3) subcutaneous fat: subcutaneous fat.

We initially included in training an additional class, “other muscle”, which comprised smaller head and neck muscle groups (Walter et al., 2024). However, after applying a brain mask–guided region-of-interest (ROI) cropping strategy (see section 2.7), this class frequently had zero visible volume. Due to its consistent exclusion from the cropped field-of-view, we opted not to evaluate or include this class in the final training or inference outputs.

### 2.2 Network training and computational requirements

TotalSegmentator is a model from the nnU-Net framework (Wasserthal et al., 2023), which is a UNet–based implementation that automatically configures all hyperparameters based on the dataset characteristics in a single-model setting. Based on the nnUNetV2 framework, the model was trained from scratch using Xavier initialization, Z-score normalization, a batch size of 2, and 128 × 128 × 128 patches. We used stochastic gradient descent with a learning rate of 1e−5 and a polynomial learning rate scheduler (exponent 0.9). The objective combined Dice and cross-entropy loss; performance was measured with pseudo-Dice. Augmentation followed nnU-Net’s automated defaults. All MRI images and their corresponding segmentations underwent quality assurance (QA), and MRIs with artifacts were excluded from the training dataset. We trained nnUNetV2 following the standard protocol, optimizing the pseudo-dice and cross-entropy for 1000 epochs without early stopping, selecting the model based on the best validation pseudo-Dice achieved. The training was conducted on an NVIDIA GeForce RTX 4090 with 24 GB VRAM, using a batch size of 2, and completed in ~8.5 hours. Inference on the same instance took 8–12 seconds per complete volume segmentation. For CPU-based inference, the minimum RAM requirement is 16 GB to accommodate the model and intermediate activations.

### 2.3 Evaluation datasets

Nine publicly available datasets were used for the evaluation (Fig. 2; Supplementary Methods 1). The CERMEP dataset (Mérida et al., 2021), which consists of 37 adults of co-registered CT-MRI pairs (45.9% (N = 17) male, mean age ± SD, 38.11 ± 11.36 years; range: 23–65 years) was used for evaluation of segmentation in healthy subjects, and the multi-center ACRIN dataset (64%(N = 29) male, mean age ± SD: 57.2 ± 9 years, range: 29–77 years), which consists of subjects with newly diagnosed glioblastoma multiforme, was used to evaluate segmentation in the setting of brain pathology (tumors). Seven additional MRI datasets (Calgary (Reynolds et al., 2020), ICBM (Kötter et al., 2001), IXI (IXI Dataset – Brain Development, n.d.), ABCD (Casey et al., 2018), PING (Rivkin et al., 2010) BabyConnectome (Howell et al., 2019), and Brats-PEDS (Kazerooni et al., 2024)) were used to evaluate TissUnet in pediatric healthy and brain tumor settings (Supplementary Methods 1). Scans were co-registered to MRI age-dependent T1-weighted asymmetric brain atlases, generated from the NIH-funded MRI Study of Normal Brain Development (NIHPD, Fonov et al., 2011), using rigid registration and rescaled to 1-mm isotropic voxel size to preserve anatomical size differences (Lasso, 2017/2023).

### 2.4 Evaluation and statistical analysis

All statistical analyses were done in R (v4.3.3). Between-group comparisons were conducted using the Mann–Whitney U test, with false discovery rate (FDR) correction for multiple comparisons. Categorical variables were compared using the Chi-Squared test. Two-sided p-values < 0.05 were considered statistically significant.

We evaluated TissUnet performance using four distinct experimental setups across nine external datasets (Fig. 2). First, we compared TissUnet-predicted segmentations of the skull, fat, and muscle to reference segmentations generated from CT using TotalSegmentator and to the GRACE method (Stolte et al., 2024) on CERMEP dataset (N = 37, Fig. 2B). All CERMEP AI-generated segmentations passed manual image QA. The performance was assessed using the Dice similarity coefficient and 95th percentile Hausdorff distance (HD95).

Second, we compared model outputs of TissUnet and GRACE (Stolte et al., 2024) to manual segmentations from an expert neuroradiologist (H.S., board-certified, 17 years of experience). We randomly selected 10 cases with paired MRI-CT imaging available, 5(50%) MRI T1w with a brain tumor (glioblastoma) from the ACRIN TCIA dataset (“ACRIN-FMISO-BRAIN,” n.d.), and 5(50%) T1w MRIs from the CERMEP dataset with no diagnosis (Mérida et al., 2021) (Fig. 2C).

Third, we conducted an acceptability assessment in which two trained annotators (A.Z., L.H.) rated segmentation quality in blinded 3D review using a 5-point Likert scale, which was categorized into “Acceptable”, “Unacceptable”, and “Bad MRI” categories (N = 108). Inter-rater agreement was quantified using Gwet AC1 (Wongpakaran et al., 2013). Disagreements were resolved by a third reviewer (B.H.K., a board-certified radiation oncologist with 9 years of experience). Subjects were randomly selected and stratified by age, sex, and dataset origin: Calgary (Reynolds et al., 2020), ICBM (Kötter et al., 2001), and IXI (IXI Dataset – Brain Development, n.d.), to ensure the diversity in imaging protocols, scanner types, and developmental stages (N = 54, 50% female, median age = 23, IQR[5–27]). To assess model performance in a pediatric brain tumor scenario, we randomly selected N = 54 patients from the BRATS-Peds 2023, which contains multi-institutional MRI scans of children diagnosed with high-grade gliomas (Fig. 2D).

Lastly, a reviewer (B.H.K.) compared TissUnet and GRACE segmentations in a blind review using Slicer 3D extension (SegmentationReview (Zapaishchykova, Tak, et al., 2023). The reviewer was blinded both to the segmentation method and diagnostic status (N = 45, Calgary (Reynolds et al., 2020), ICBM (Kötter et al., 2001), IXI (IXI Dataset – Brain Development, n.d.), ABCD (Casey et al., 2018), PING (Rivkin et al., 2010), BabyConnectome (Howell et al., 2019) Brats-PEDS (Kazerooni et al., 2024)), Fig. 2E).

We conducted two ablation studies. In the first one, to test brain ROI robustness we simulated the imperfect registration by applying a ±5-degree tilt, using the same dataset described above used for the blinded 3D review (Fig. 2D, N = 108, where N = 54 patients from the BRATS-Peds 2023 and N = 54 healthy subjects randomly selected and stratified by age, sex, and dataset origin from three datasets: Calgary, ICBM and IXI). In the second ablation study, we evaluated the model’s robustness to intensity variations by applying contrast and brightness augmentations (gamma adjustments of 0.7 and 1.5, brightness shifts of ±0.2, and intensity scaling to 0.0–1.2 range) to the same dataset and comparing the resulting volumetric segmentations.

To assess associations between blood cholesterol levels and predictors—including BMI, extracranial tissue volumes, sex, and age—we used uni- and multivariable linear regression models. The Box-Cox transformation was applied to normalize the cholesterol distribution. Model assumptions, including linearity, normality of residuals, homoscedasticity, and absence of multicollinearity, were evaluated using diagnostic plots, the Shapiro–Wilk test, and variance inflation factors.

### 2.5 Application of TissUnet-derived volumetrics as predictors of cholesterol

To demonstrate potential clinical application, we used the Adolescent Brain Cognitive Development (ABCD) Study (Casey et al., 2018), a large-scale, multi-institutional cohort designed to investigate brain and health development across adolescents. We applied TissUnet-derived tissue volumes to model relationships between muscle, fat, BMI, and blood cholesterol levels in youth. We compared univariable and multivariable linear regression models. The outcome variable, total blood serum cholesterol, was Box-Cox transformed (λ = 0.2) to approximate normality and stabilize the variance. Predictors included Body Mass Index (BMI), volumetric measures of the muscle and subcutaneous fat (derived from TissUnet), sex, and age. Assumptions of linearity, normality of residuals, homoscedasticity, and multicollinearity were assessed through diagnostic plots and variance inflation factors.

### 2.6 Skull thickness estimation

Skull thickness is typically calculated via CT, but is comparatively difficult to assess via MRI, given poorer bone-tissue contrast. For TissUnet-based skull thickness estimation, we extracted the contour from the TissUnet predicted bone mask using OpenCV Python (Fig. 1A). We calculated median skull thickness by aggregating the central 95% of values (i.e., between the 2.5–97.5 percentiles) from over 100 tangents in each of 16 consecutive 1 mm 2D axial slices, beginning 10 mm superior to the top orbital roof detected by the DL method (Zapaishchykova, Liu, et al., 2023) to offset frontal sinus cavities and orbital cavities. We compared the TissUnet median MRI skull thickness to CT-based thickness as a reference standard (default threshold set at 471 HU (Delso et al., 2015). We also compared performance across four other MRI-based methods for skull detection (GRACE (Stolte et al., 2024), CHARM (Puonti et al., 2020), BrainSuite (Shattuck & Leahy, 2002), and SPM25 (Friston et al., 2006; Tierney et al., 2025)).

### 2.7 Brain ROI

To account for the variability in defacing and the different ROI between MRI scanners across various open-source datasets, we introduce the brain mask-based ROI area cropping pipeline. It allows us to standardize the measurement area of anatomical regions for comparison between subjects. Briefly, we first identify where the brain mask begins in each slice, then cut away everything “in front” of that point. This creates a consistent, standardized brain region across different datasets and scanners, regardless of how the data were defaced originally.

Brain masks were generated using HD-BET (Isensee et al., 2019). For each axial slice, we identified the most anterior point of the brain mask and used it as a dynamic cut-off line. All voxels anterior to this point were removed, retaining only the region posterior to the boundary as the ROI. Formally, consider a 3D MRI volume $V\in R^{H\;\;\times \;W\;\;\times \;\;D},$ where *H, W,* and *D* correspond to the height (posterior-anterior axis), width (left-right axis), and depth (inferior-superior axis), respectively. The associated binary brain mask $S\in {\left\{0,1\right\}}^{H\;\;\times \;\;W\text{​}\;\times \;\;D}$ indicates whether each voxel belongs to brain tissue:

$$\begin{array}{c} S(x,y,z)=\{\begin{array}{c} \begin{array}{l} 1,if\;the\;voxel\;at\;coordinate\;(\text{x},\text{y},\text{z})\;is\;\\ classified\;as\;brain\;tissue,\\ 0,\;otherwise\text{​} \end{array} \end{array}\; \end{array}$$ (1)

Processing proceeds slice by slice in the transverse (XY) plane, from inferior (z = 0) to superior (z = D−1). For each slice z, the top point T(z) represents the most anterior voxel where brain tissue is present:

$$\begin{array}{l} T(z)=\max\text{\{}x\in [0,H-1]\;\text{|}\;\;\exists \;y\;\in [0,W-1]\;such\;that\;\\ S(x,y,z)=1\}\;, \end{array}$$ (2)

If a slice contains no brain tissue, T(z)=0.

To maintain a consistent defacing boundary across slices, we define an adjusted top point T*(z) that propagates the most anterior boundary observed so far:

$$\begin{array}{c} T*\left(z\right)=\max\{T(z),T^{*}(z-1)\},\;\;with\;\;T*(-1)=0 \end{array}$$ (3)

This prevents the cutting plane from moving posteriorly in subsequent slices. Using

T*(z), we update the brain mask to remove voxels anterior to the adjusted boundary:

$$\hat{S}(x,y,z)=\{\begin{array}{c} 0,if\;x>T*(x),\text{​}\\ S(x,y,z),\;otherwise \end{array}\;$$ (4)

The same rule is applied to the original MRI volume to obtain the defaced image:

$$\hat{V}(x,y,z)=\{\begin{array}{c} 0,if\;x>T*(x),\text{​}\\ V(x,y,z),\;otherwise \end{array}\;$$ (5)

**Fig. 3.**
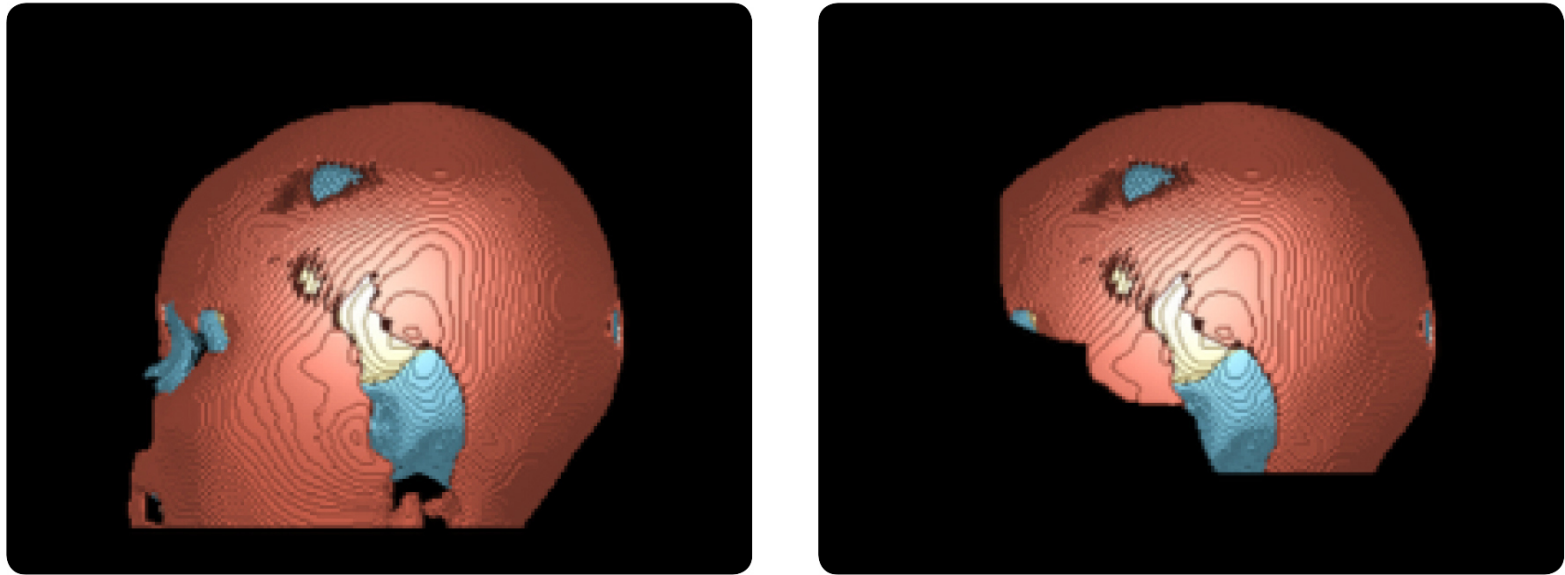
Example of brain ROI cropping. The left panel shows a sagittal view of an original MRI scan and predictions, while the right panel displays the defaced counterparts.

By zeroing out voxels anterior to T*(z), facial features are effectively removed while the brain regions necessary for downstream analysis are preserved (Fig. 3).

## 3 Results

### 3.1 Segmentation evaluation

**Fig. 4.**
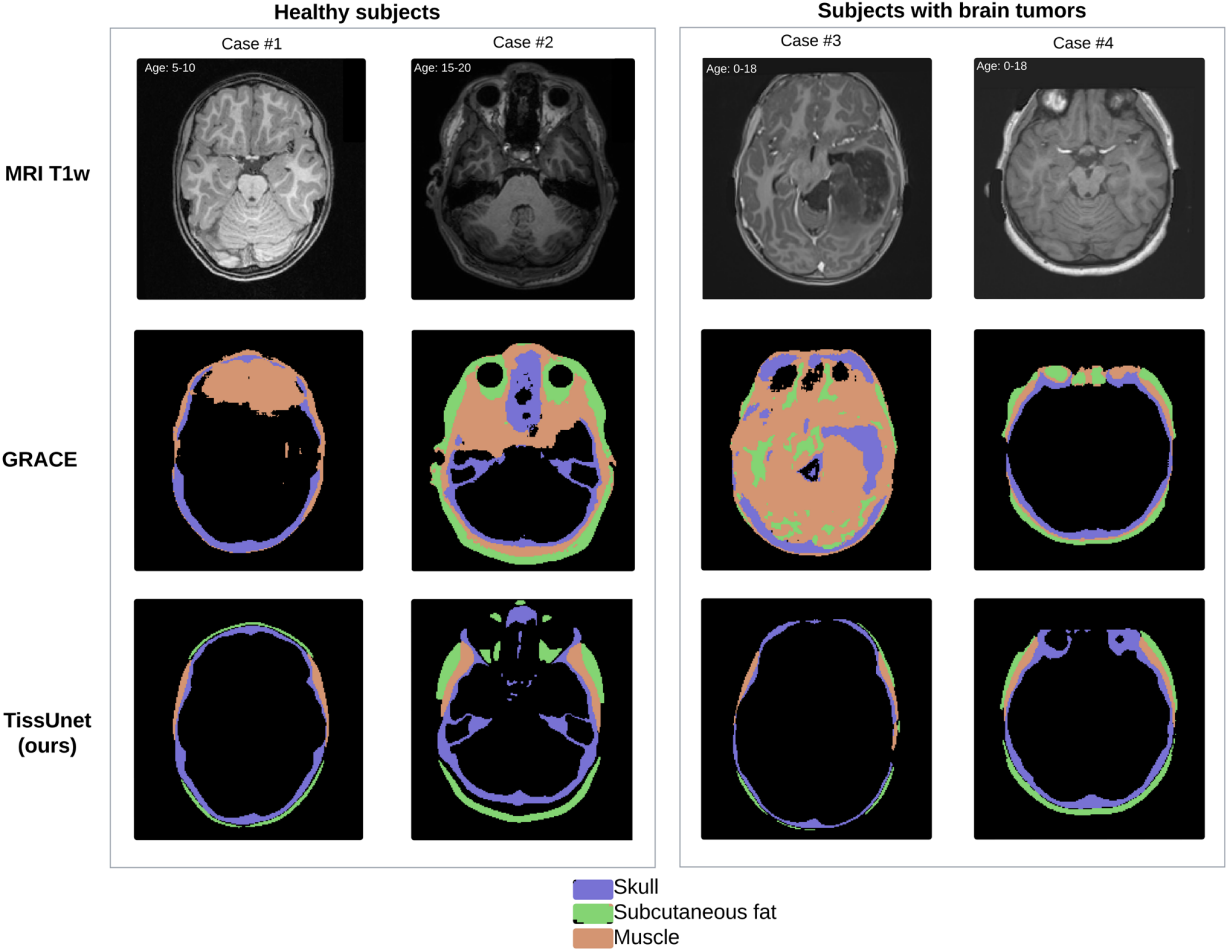
Example inference from the T1-MRI. The results are shown in axial view. We compared GRACE (MRI T1w) (Stolte et al., 2024) to TissUnet (our) model in different age groups (overlaid in white text) and different health statuses: healthy (cases #1 and #2), and subjects with brain tumors (cases #3 and #4). Skull mask in purple, muscle in orange, and subcutaneous fat in green.

**Fig. 5.**
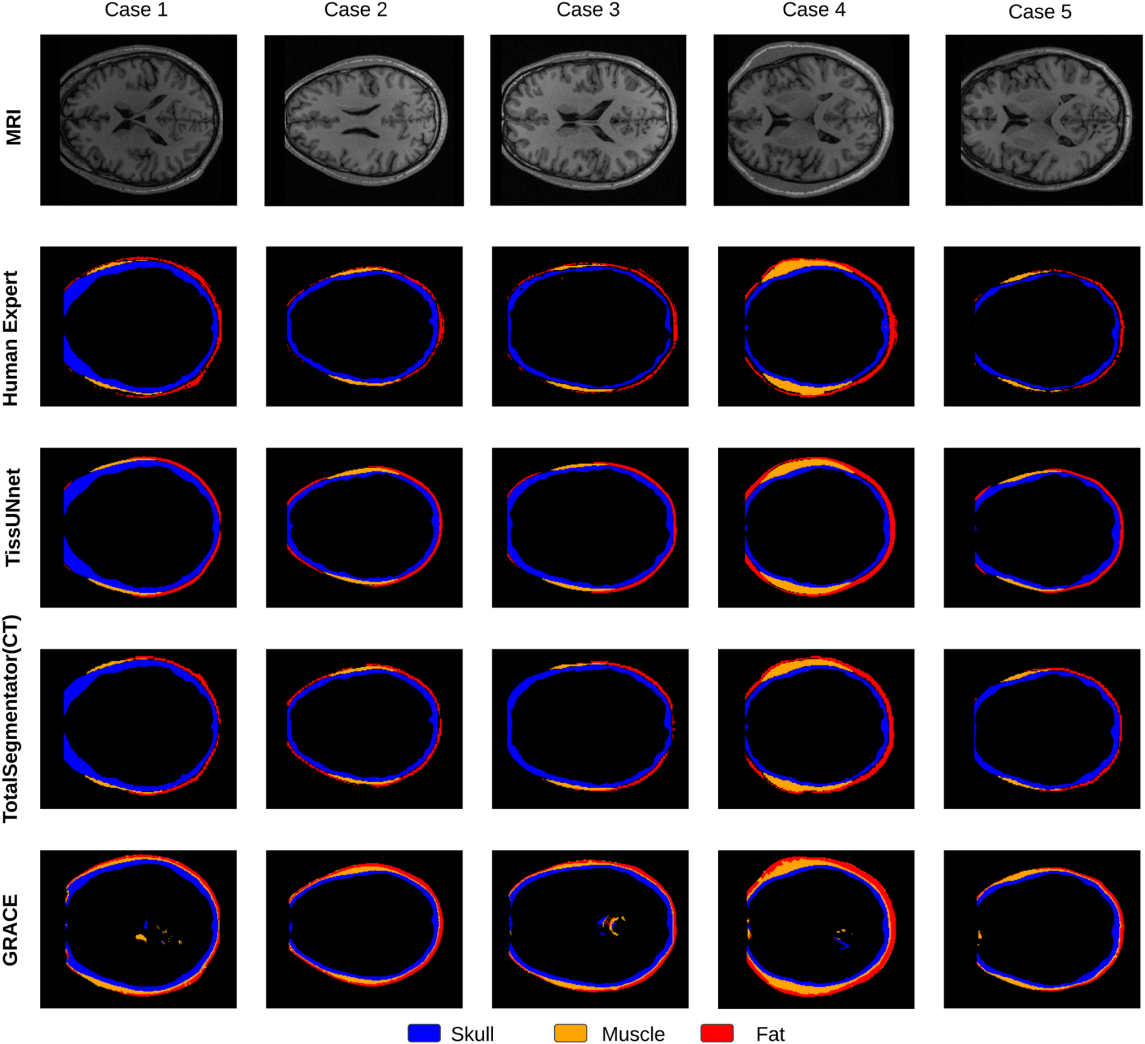
Sample of five cases segmentations for three classes (skull in blue, muscle in yellow, and subcutaneous fat in red) from the T1-MRI. The results are shown in axial view, slice z* = *100. We compared GRACE (MRI T1w) to TissUnet (our), Human Expert annotations, and TotalSegmentator (predicted over CT image).

We manually reviewed each sample in the SynthRad training dataset and removed 13.8% of T1-weighted MRIs (N = 25) due to imaging artifacts, including motion and blurring. In the AI-CT as ground-truth validation study, TissUnet median Dice in the external cohort of healthy adult subjects was 0.79 [IQR 0.77–0.81], compared to 0.5[0.48–0.54] for GRACE (p < 0.001) with significant improvements in skull, fat, and muscle segmentation (Fig. 1B, Table 1). In the second validation study using manual human expert annotations as ground truth, TissUnet’s median Dice in the external cohort of healthy subjects was 0.83 [IQR: 0.83–0.84] and in the cohort with brain tumors was 0.81 [IQR: 0.78–0.83] (Fig. 1C, Table 2), compared to 0.73[0.7–0.74] and 0.6[0.57–0.62] for GRACE, respectively. TissUnet showed consistently higher segmentation accuracy across different groups (see Fig. 4, Fig. 5 for example segmentations).

**Table 1.** Median dice and HD95 [IQR] on 3 tissue classes and overall, comparing GRACE and TissUnet models vs. AI-CT annotations on N = 37 cases from the CERMEP dataset (Mérida et al., 2021).

	Dice, median [IQR] ↑				HD95, median [IQR] ↓			
Model	Skull	Fat	Muscle	Overall	Skull	Fat	Muscle	Overall
TissUnet (ours)	0.87 [0.85, 0.88]	0.65 [0.58, 0.68]	0.86 [0.84, 0.88]	**0.79** **[0.77, 0.81]**	3.79 [3.53, 4.35]	2.53 [2.22, 2.91]	3.53 [3.08, 3.93]	**3.35** **[3.16, 3.61]**
GRACE	0.78 [0.77, 0.79]	0.49 [0.44, 0.52]	0.25 [0.21, 0.29]	**0.50** **[0.48, 0.54]**	3.61 [3.3, 3.95]	2.24 [2.12, 2.34]	3.06 [2.67, 3.41]	**3.02** **[2.78, 3.25]**

IQR = interquartile range, HD95 = The 95th percentile Hausdorff Distance.

**Table 2.** Median, IQR dice, and HD95 on 3 tissue classes, comparing GRACE and TissUnet models vs. human expert annotations on N = 10 cases (N = 5 brain tumor and N = 5 healthy subjects).

	Dice, median [IQR] ↑					HD95, median [IQR] ↓			
Health Status	Model	Skull	Fat	Muscle	Overall	Skull	Fat	Muscle	Overall
Healthy	TissUNet (ours)	0.83 [0.83, 0.86]	0.59 [0.58, 0.6]	0.84 [0.84, 0.87]	0.83 [0.83, 0.84]	1.41 [1, 1.73]	3.46 [3, 3.74]	1 [1,1.41]	1.73 [1.41, 1.73]
	GRACE	0.76 [0.75, 0.76]	0.73 [0.7, 0.74]	0.18 [0.16, 0.27]	0.73 [0.7, 0.74]	5.1 [3, 5.48]	6.71 [6.48, 7.14]	87.07 [75.05, 89.99]	6.71 [6.48, 7.14]
Brain tumor	TissUNet (ours)	0.9 [0.89, 0.91]	0.72 [0.7, 0.73]	0.81 [0.78, 0.83]	0.81 [0.78, 0.83]	1 [1,1]	5 [3, 19.52]	1.73 [1.73, 2.24]	1.73 [1.73, 2.24]
	GRACE	0.74 [0.71, 0.76]	0.6 [0.57, 0.62]	0.2 [0.18, 0.2]	0.6 [0.57, 0.62]	3.74 [3, 5.38]	20.4 [13.42, 28.05]	85.15 [81.8, 88.92]	20.4 [13.42, 28.05]

IQR = interquartile range, HD95 = The 95th percentile Hausdorff Distance.

In the acceptability testing, following adjudication by a tiebreaker for cases with disagreement, the final acceptability rates were 89% (N = 289) “Acceptable,” 10% (N = 34) “Unacceptable,” and 0.1% (N = 1) “Bad Images” (Fig. 1D). Agreement between the Tie Breaker with Reviewer 1 was Gwet AC1 of 0.89 (95% CI: 0.86–0.93) and with Reviewer 2 Gwet AC1 of 0.72 (95% CI: 0.65–0.78). Intra-rater agreement was higher in the healthy cohort compared to the brain tumor cohort across all tissue types (Table 3).

In the blinded review, TissUnet had 100% (N = 45) of cases rated as acceptable, whereas GRACE had 16% rated as acceptable, with 84% (N = 38) labeled as requiring revision or edits (Fig. 1E).

**Table 3.** Inter-rater agreement in acceptability testing on 3 tissue classes (skull, subcutaneous fat, and muscle) was measured using Gwet AC1(with 95 %CI in parentheses) within N = 108 subjects (N = 54 patients with brain tumor and N = 54 healthy subjects).

Health status	Rater	Skull	Fat	Muscle	Overall
Brain tumor	R1-R2 R1-TB R2-TB	0.200 [-0.005, 0.405] 0.649 [0.443, 0.855] 0.457 [0.259, 0.654]	0.699 [0.542, 0.856] 0.920 [0.838, 1.000] 0.791 [0.660, 0.921]	0.751 [0.588, 0.913] 0.981 [0.943, 1.000] 0.777 [0.625, 0.929]	0.573 [0.470, 0.676] 0.878 [0.822, 0.935] 0.687 [0.596, 0.778]
Healthy	R1-R2 R1-TB R2-TB	0.371 [0.166, 0.576] 0.774 [0.635, 0.912] 0.462 [0.264, 0.660]	0.798 [0.671, 0.925] 0.943 [0.876, 1.000] 0.852 [0.734, 0.971]	0.852 [0.734, 0.971] 1.000 [1.000, 1.000] 0.852 [0.734, 0.971]	0.693 [0.604, 0.783] 0.913 [0.865, 0.961] 0.742 [0.658, 0.826]

R1 = Rater 1, R2 = Rater 2, TB = Tie Breaker.

### 3.2 Skull thickness methods comparison

**Fig. 6.**
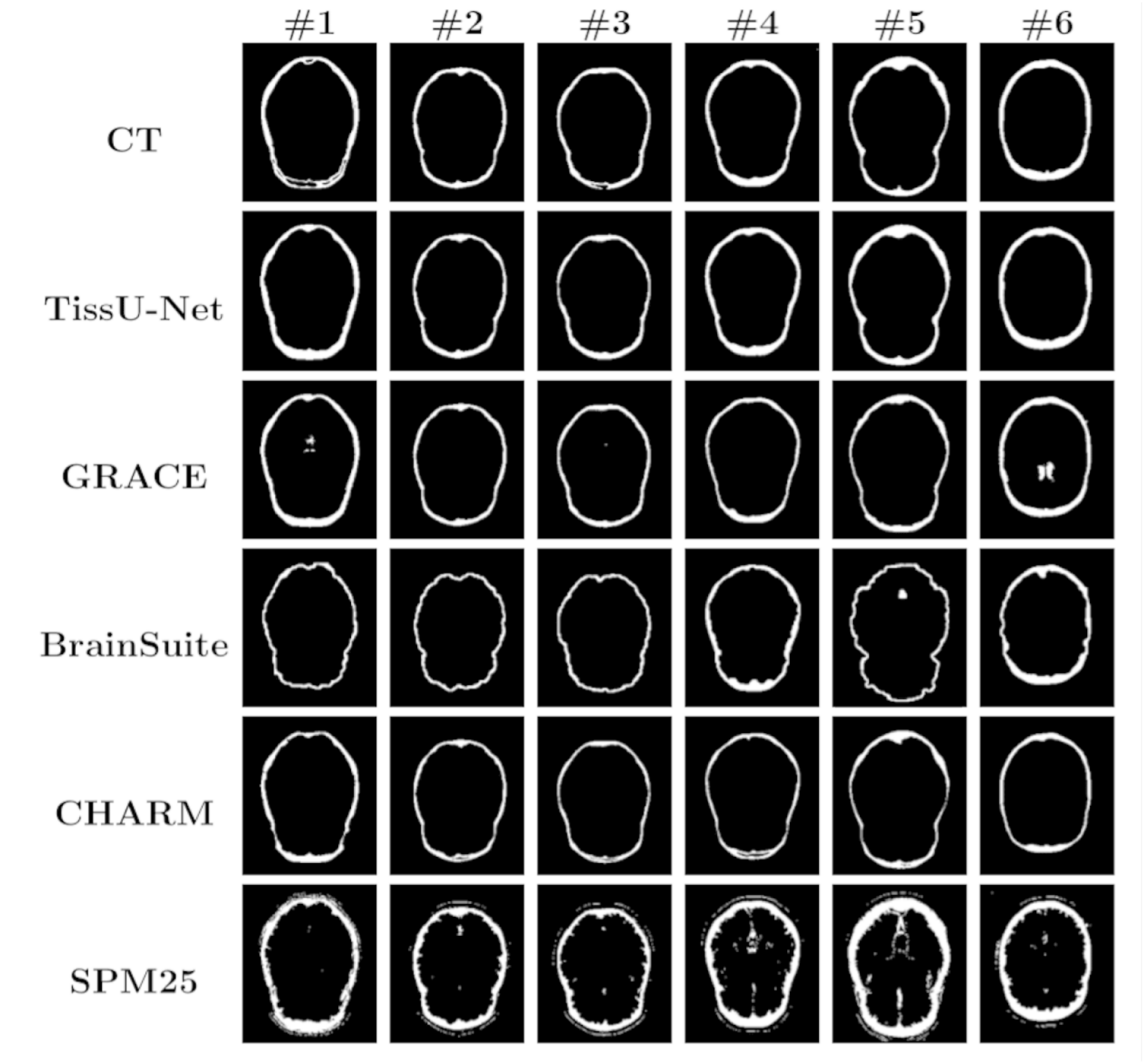
Skull segmentations of three randomly selected healthy participants (columns #1-#3) from the CERMEP dataset and three randomly sampled patients with brain tumors (columns #4-#6) from ACRIN-TCIA (axial view) for CT (HU>471 (Delso et al., 2015)) and T1w MRI-based segmentation models (TissUnet, GRACE (Stolte et al., 2024), CHARM (Puonti et al., 2020), BrainSuite (Shattuck & Leahy, 2002), SPM25 (Friston et al., 2006; Tierney et al., 2025)).

TissUNet yielded skull thickness measurements that more closely aligned with CT-based thickness than other MRI-based approaches both in the healthy and brain tumor patient cohorts at the default HU threshold (Table 4 and Fig. 6) and across various CT HU window settings.

**Table 4.** Skull thickness mean thickness and absolute difference (in mm) in healthy cohort CERMEP (N = 37), add brain tumor cohort ACRIN (N* = *5) for CT (reference, HU>471 (Delso et al., 2015)), TissUnet (our method), GRACE (Stolte et al., 2024), CHARM (Puonti et al., 2020), BrainSuite (Shattuck & Leahy, 2002), and SPM25 (Friston et al., 2006; Tierney et al., 2025).

	Healthy		With brain tumor	
Tool	Mean thickness (SD)	Absolute difference (Tool – CT)	Mean thickness (SD)	Absolute difference (Tool – CT)
CT (Reference)	5.60 (0.81)	-	6.81 (1.17)	-
TissUNet (ours)	5.46 (0.90)	0.31	7.38 (1.22)	0.57
GRACE	5.05 (0.71)	0.60	5.61 (1.07)	1.21
CHARM	4.48 (0.69)	1.11	3.88 (0.84)	2.93
BrainSuite	3.09 (0.79)	2.51	5.74 (2.61)	1.48
SPM25	8.01 (1.48)	2.52	9.93 (1.98)	3.11

SD = standard deviation, HU = Hounsfield Units, CT = computer tomography.

**Fig. 7.**
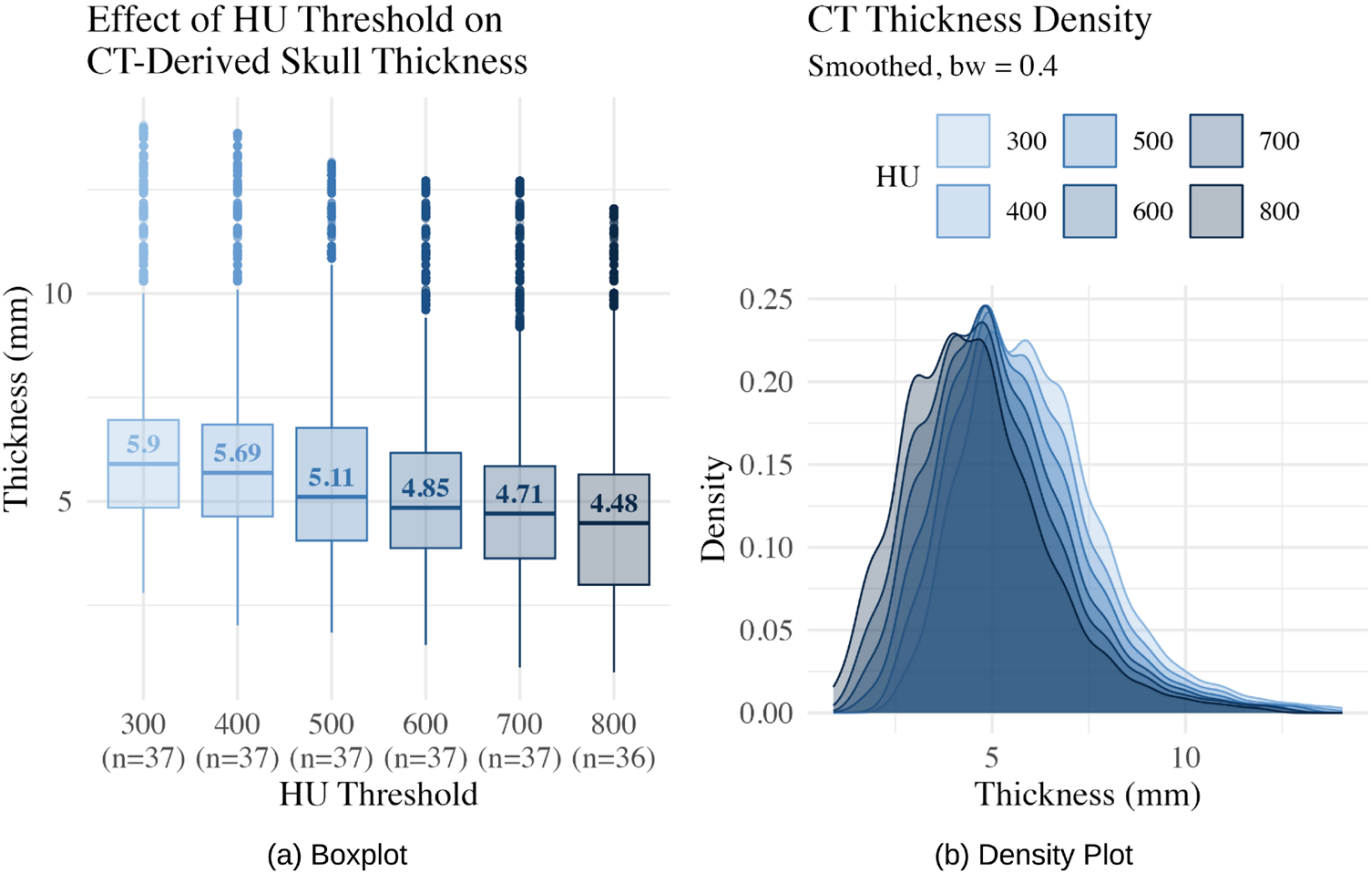
Boxplot (a) and density plot (b) showing the distribution of median skull thickness measured from CT across different Hounsfield Unit (HU) threshold windows (300–800 HU) in the CERMEP dataset (N = 37 healthy subjects). One subject was excluded from the HU = 800 threshold due to a higher density skull and absence of a valid contour.

**Fig. 8.**
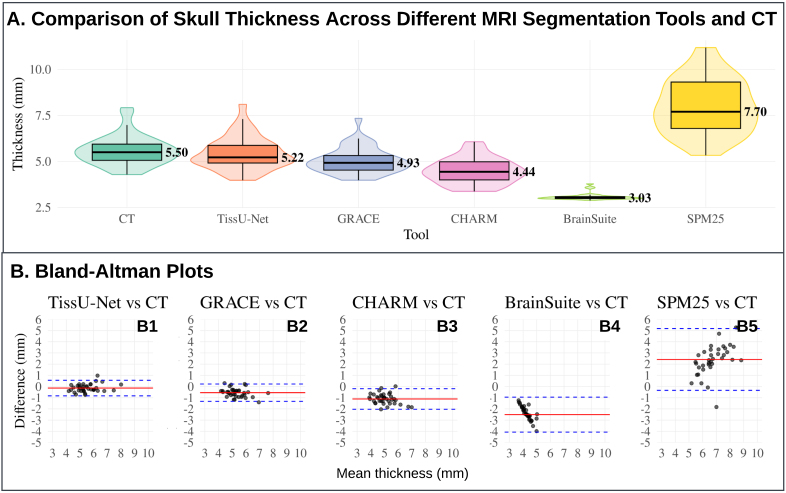
(A) Comparison of median skull thickness between CT (reference, HU > 471 (Delso et al., 2015)), TissUnet, GRACE (Stolte et al., 2024), CHARM (Puonti et al., 2020), BrainSuite (Shattuck & Leahy, 2002), and SPM25 (Friston et al., 2006; Tierney et al., 2025). The CERMEP dataset includes 37 healthy subjects with paired MRI T1-weighted (T1w) and CT images. The violin plots illustrate the overall distribution shape, with overlaid boxplots indicating the median (also labeled in text) and quartiles (see Table 4). (B) Bland–Altman plots compare each MRI-based segmentation tool (TissUnet, GRACE, CHARM, BrainSuite, SPM25) to the CT reference on the CERMEP dataset (Mérida et al., 2021). The solid red line represents the mean difference (bias), and the dashed blue lines indicate the 95% limits of agreement. CT = Computed Tomography, HU = Hounsfield units.

Skull thickness was estimated from computed tomography (CT) using HU-based thresholding to segment cranial bone. To evaluate the sensitivity of skull thickness measurements to different segmentation thresholds, we compared multiple HU window settings: 300, 400, 500, and 800 HU on the CERMEP dataset (N = 37 healthy adult subjects, see Fig. 7). These thresholds reflect the range of expected attenuation values for cancellous and cortical bone, which vary with age and bone mineral density (Delso et al., 2015; Schulte-Geers et al., 2011). Lower thresholds (e.g., 300 HU) are more inclusive of less mineralized or spongy bone typically seen in pediatric populations, whereas higher thresholds (e.g., 800 HU) emphasize dense cortical bone but may exclude thinner or under-ossified regions. Pediatric studies report skull attenuation values increasing from approximately 450–550 HU at birth to ~1100 HU by age 10, while adult cortical bone typically exhibits values of ≥900–1000 HU (Delso et al., 2015; Schulte-Geers et al., 2011). In brain tumor patients, especially those with peritumoral brain edema, skull HU tends to be lower than in matched healthy or non-edematous controls, particularly in older age groups (Kang et al., 2021). We excluded one subject from the study with 800 HU due to anomalously high skull density and the absence of a valid segmentation contour at that threshold. TissUnet demonstrated excellent agreement across a range of skull thickness magnitudes, with a mean difference of 0.31 mm in the healthy and 0.57 mm in the brain tumor cohort (Fig. 8).

### 3.3 Ablation studies

When simulating minor registration errors by applying a ±5-degree tilt, estimated tissue volumes remained stable, with absolute average percentile changes of less than 3% across all classes and health groups (Table 5). Based on a sample size of 54 participants per group, the study had 94.6% power to detect a moderate effect size (Cohen’s *d* ≈ 0.5) between groups using a two-sided Wilcoxon signed-rank test (two-sided 0.05 type I error).

**Table 5.** Average volumetric differences (in cm² and as percentiles relative to the unmodified volume (“zero” tilt) mean ± SD) following simulated ±5° head rotations (forward and backward) across 108 MRI (Healthy: n = 54; Brain tumor: n = 54).

Health status	Tilt angle	Skull	Fat	Muscle
Brain tumor	+5	684.81 ± 563.88 0.23% ± 0.17% (p = 0.95)	853.24 ± 736.78 0.78% ± 0.70% (p = 0.94)	37.54 ± 50.70 0.13% ± 0.16% (p = 0.99)
	-5	1493.69 ± 1258.68 0.50% ± 0.38% (p = 0.85)	1128.59 ± 1352.88 1.04% ± 1.00% (p = 0.86)	193.41 ± 178.75 0.70% ± 0.76% (p = 0.81)
Healthy	+5	1054.83 ± 933.30 0.28% ± 0.21% (p = 0.99)	2453.22 ± 1454.75 2.10% ± 1.32% (p = 0.79)	35.74 ± 48.98 0.09% ± 0.10% (p = 0.97)
	-5	2260.83 ± 1672.91 0.64% ± 0.44% (p = 0.78)	3580.98 ± 2119.41 3.19% ± 2.06% (p = 0.74)	311.24 ± 296.54 0.79% ± 0.64% (p = 0.79)

Pairwise group differences were assessed using a two-sided Wilcoxon–Mann–Whitney U-test.

SD = standard deviation.

We evaluated model robustness to intensity variations by applying five augmentations using MONAI transforms: high contrast (gamma = 1.5), low contrast (gamma = 0.7), brightness increase (+0.2 offset), brightness decrease (-0.2 offset), and intensity scaling (0.0–1.2 range). We compared differences in estimated volumetrics intrasubject, with average percentile changes of less than 1% across all classes and health groups, and a maximum change of 2.4% observed for fat tissue under low contrast conditions (Table 6).

**Table 6.** Ablation study of contrast augmentation effects on extracranial tissue segmentation.

Health status	Augmentation	Skull	Fat	Muscle
Healthy	No augmentation	369.205 ± 102.796	142.904 ± 85.674	42.789 ± 22.802
	High contrast	367.970 Δ=-1.2345 cm³ (-0.33%)	141.613 Δ=-1.2909 cm³ (-0.90%)	42.643 Δ=-0.1456 cm³ (-0.34%)
	Low contrast	369.598 Δ=+0.3939 cm³ (+0.11%)	144.792 Δ=+1.8872 cm³ (+1.32%)	43.638 Δ=+0.8498 cm³ (+1.99%)
	Brightness increase	369.132 Δ=-0.0725 cm³ (-0.02%)	142.823 Δ=-0.0814 cm³ (-0.06%)	43.002 Δ=+0.2135 cm³ (+0.50%)
	Brightness decrease	369.132 Δ=-0.0722 cm³ (-0.02%)	142.823 Δ=-0.0812 cm³ (-0.06%)	43.002 Δ=+0.2137 cm³ (+0.50%)
	Intensity scaled	369.133 Δ=-0.0719 cm³ (-0.02%)	142.823 Δ=-0.0810 cm³ (-0.06%)	43.002 Δ=+0.2132 cm³ (+0.50%)
Cancer	No augmentation	293.399 ± 84.793	129.616 ± 68.420	32.357 ± 20.111
	High contrast	292.580 Δ=-0.8186 cm³ (-0.28%)	128.500 Δ=-1.1164 cm³ (-0.86%)	32.496 Δ=+0.1394 cm³ (+0.43%)
	Low contrast	291.091 Δ=-2.3085 cm³ (-0.79%)	134.291 Δ=+4.6746 cm³ (+3.61%)	32.867 Δ=+0.5098 cm³ (+1.58%)
	Brightness increase	291.982 Δ=-1.4170 cm³ (-0.48%)	131.809 Δ=+2.1924 cm³ (+1.69%)	32.660 Δ=+0.3030 cm³ (+0.94%)
	Brightness decrease	291.994 Δ=-1.4048 cm³ (-0.48%)	131.810 Δ=+2.1934 cm³ (+1.69%)	32.660 Δ=+0.3031 cm³ (+0.94%)
	Intensity scaled	291.982 Δ=-1.4169 cm³ (-0.48%)	131.807 Δ=+2.1908 cm³ (+1.69%)	32.660 Δ=+0.3032 cm³ (+0.94%)

Volumetric measurements (cm³, mean ± SD) for skull, subcutaneous fat, and temporalis muscle across different image augmentations (N = 54 patients with brain tumor and N = 54 healthy subjects). Augmentations were applied using MONAI transforms and included: high contrast (gamma = 1.5), low contrast (gamma = 0.7), brightness increase (offset = +0.2), brightness decrease (offset = -0.2), and intensity scaling (range 0.0–1.2). Augmented results show mean volume with absolute (Δ cm³) and relative (%) differences compared to non-augmented baseline.

### 3.4 Application of TissUnet-derived volumetrics as predictors of cholesterol

In the ABCD study, 888 subjects had blood cholesterol and corresponding T1w MRI available. Median age was 11.9 years [IQR 11.3–12.4], 44% (N = 389) female, median BMI 19.2[IQR 17.2–22.6], median blood cholesterol was 155 (mg/dL) [IQR 139–173]. 71% (N = 631) of subjects had normal cholesterol (less than 170 mg/dL (Hyperlipidemia in Children | Symptoms, Diagnosis & Treatment, n.d.)). Median extracranial muscle volume was 39 cm^3^ [IQR 33–47], and median extracranial subcutaneous fat volume was 110 cm^3^ [IQR 79–172].

**Fig. 9.**
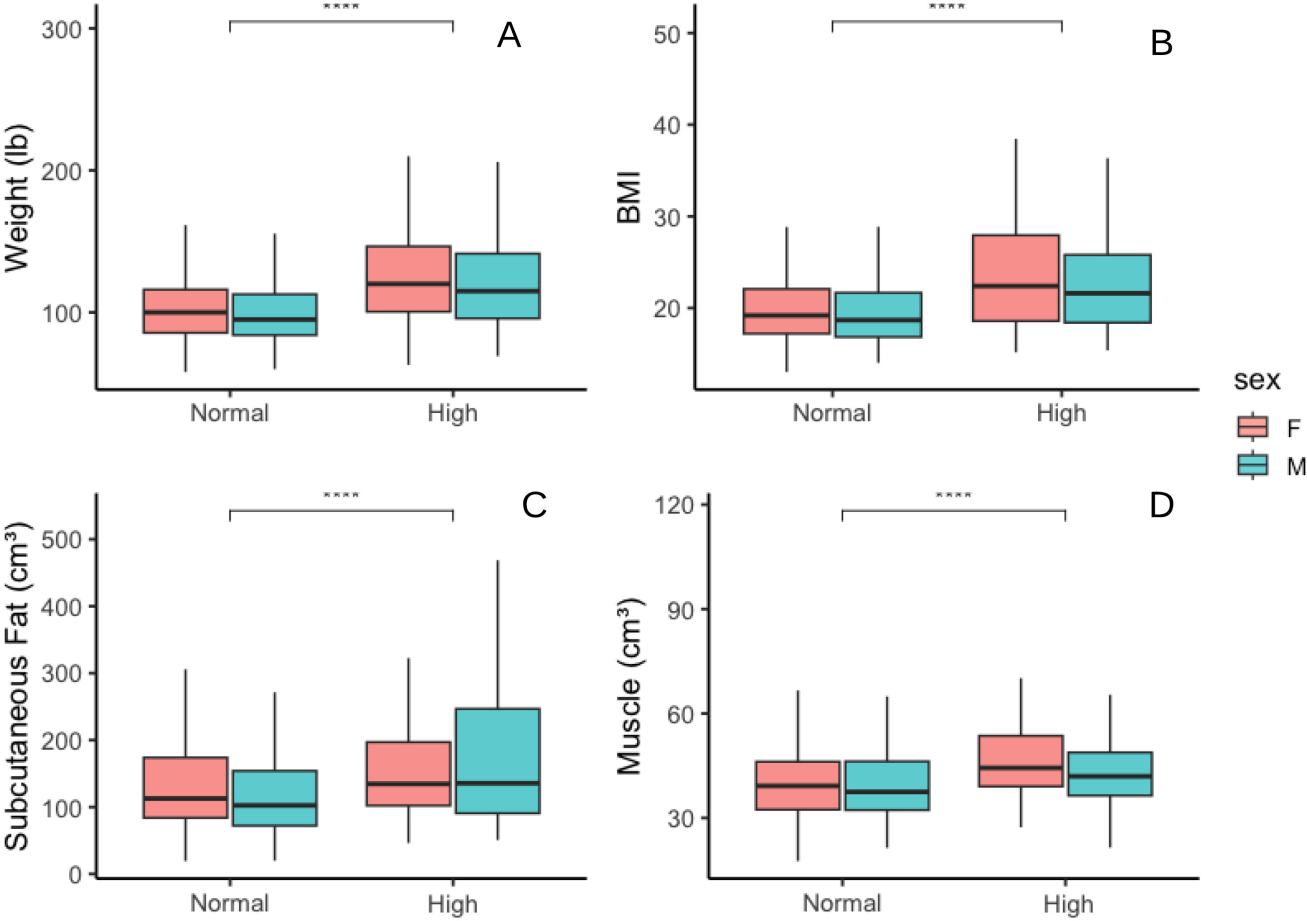
Associations between blood cholesterol levels and body composition metrics (BMI and weight) and extracranial volumetrics (muscle and subcutaneous fat), stratified by sex (N = 888 subjects). Boxplots show distributions of weight (A), BMI (B), subcutaneous fat volume in cm^3^ (C), and temporalis muscle volume in cm^3^ (D) across binary cholesterol categories (below 170 mg/dl is “Normal” and above is “High”). Boxes represent the interquartile range (IQR), with the median shown as a horizontal line; whiskers extend to 1.5× IQR. Statistical comparisons between cholesterol groups were done using a two-sided Mann–Whitney U test with FDR adjustment for multiple comparisons. Asterisks indicate significance levels: p < 0.0001 (****); ns = not significant. IQR = interquartile range, BMI = body mass index.

Temporalis muscle volume was significantly associated with blood cholesterol in the multivariable regression model, adjusted for age, sex, and subcutaneous fat (β = –4.23 × 10^-3^, p = 0.014, Fig. 9, Tables 7–9 for uni- and multivariable models).

**Table 7.** Univariable linear models for Box-Cox transformed cholesterol.

Predictor	Estimate	95% CI	p-value
BMI	0.0026	(-0.004, 0.0092)	0.44
Muscle (cm^3^)	-0.0039	(-0.0067, -0.0012)	0.0054
Subcutaneous fat (cm³)	-0.0001	(-5e-04, 3e-04)	0.54
Sex, male	-0.0496	(-0.1123, 0.0131)	0.12
Age	-0.0053	(-0.0093, -0.0013)	0.0089

**Table 8.** Multivariable linear model for Box-Cox transformed cholesterol.

Predictor	Estimate	95% CI	p-value
BMI	0.0086	(-0.0017, 0.0189)	0.1
Muscle (cm^3^)	-0.0042	(-0.0076, -9e-04)	0.014
Subcutaneous fat (cm³)	-0.0003	(-8e-04, 2e-04)	0.27
Sex, male	-0.0331	(-0.0995, 0.0334)	0.33
Age	-0.0059	(-0.0103, -0.0015)	0.0081

**Table 9.** Median and IQR by sex and cholesterol group (below 170 mg/dl is “Normal” and above is “High”), N = 888 subjects (Fig. 9).

Sex	Cholesterol level		n	Median	IQR
F	Normal	BMI	387	19.1900	4.88000
F	High	BMI	68	22.4000	9.35750
M	Normal	BMI	462	18.6850	4.82750
M	High	BMI	103	21.6100	7.39500
F	Normal	Weight, lbs	387	100.0000	30.47500
F	High	Weight, lbs	68	120.0500	46.00000
M	Normal	Weight, lbs	462	95.0000	28.77500
M	High	Weight, lbs	103	115.0000	45.65000
F	Normal	Subcutaneous fat (cm³)	329	112.7250	90.02500
F	High	Subcutaneous fat (cm³)	60	134.5030	94.83625
M	Normal	Subcutaneous fat (cm³)	405	102.4660	81.94600
M	High	Subcutaneous fat (cm³)	94	135.5530	156.07050
F	Normal	Muscle (cm^3^)	329	39.1980	13.77100
F	High	Muscle (cm^3^)	60	44.3705	14.55250
M	Normal	Muscle (cm^3^)	405	37.4760	13.97900
M	High	Muscle (cm^3^)	94	41.9515	12.40125

## 4 Discussion

In this study, we present TissUnet, a deep learning-based model for automated segmentation of extracranial tissues—skull, subcutaneous fat, and muscle—from T1-weighted brain MRI. Compared to previously proposed methods (Stolte et al., 2024), which focused on broad tissue segmentation in adults, TissUnet was validated across the entire lifespan, from pediatric through elderly cohorts, as well as in patients with brain tumors. By leveraging pseudo-labels derived from the AI-CT-based segmentation method (Wasserthal et al., 2023), we mitigated the need for time-consuming, large-scale manual MRI annotations. TissUnet achieved high agreement with both AI-CT-based labels and human experts on five external datasets, demonstrating generalizability across different age groups and pathologies. The model achieved median Dice scores of 0.81 and 0.83 in healthy and brain tumor cohorts, respectively. We extend the TissUnet beyond segmentation and volumetric calculation of extracranial tissues, and propose an automated skull thickness estimation pipeline, broadening the utility for applications in cranial growth tracking and surgical planning.

While CT remains the clinical reference for skull thickness estimation due to its calibrated HU values, median skull HU decreases with age, from 800–850 HU in younger adults to 500–600 HU in older individuals (Delso et al., 2015; Schulte-Geers et al., 2011), our automated TissUnet-based skull thickness pipeline does not rely on specific HU thresholds and produces measurements comparable to those derived from CT across the lifespan. This approach enables accurate and efficient estimation on MRI, which is heavily utilized for tracking health conditions, such as cancer and neurologic diseases, due to its superior soft tissue contrast and absence of radiation exposure when compared to CT.

We found that TissUnet performed better across tissue segmentation tasks, particularly in pediatric and tumor cases, when directly compared to previous methods in both quantitative metrics and blinded clinical acceptability evaluation. In blinded review, all TissUnet outputs were rated acceptable, while 84% of segmentations from the previous state-of-the-art method required revision. We believe its superior performance stems from two key factors. First, the training data: compared to the previous method, which was trained exclusively on older adults from a single site, our model was trained on the multi-center SynthRAD2023 dataset, spanning a wider age range and greater anatomical variability. Second, the use of nnUNetV2, which incorporates advanced automated augmentation and is more robust to variations in MRI protocols, reducing the need for extensive image normalization. Notably, training on cases with pathologies did not impair performance on healthy brains. We hypothesize that many subtle pathologies resemble normal anatomy. In skull thickness estimation, traditional methods such as CHARM (Puonti et al., 2020), BrainSuite (Shattuck & Leahy, 2002), and SPM25 (Friston et al., 2006; Tierney et al., 2025) rely on geometric or probabilistic assumptions and skull surface meshes that often fail in the presence of abnormal anatomy. For instance, SPM125 uses voxel-wise statistical models, BrainSuite applies morphology-based techniques, and CHARM employs a mesh-based probabilistic atlas. FreeSurfer (FreeSurfer Developers, 2018), though widely used, does not explicitly segment the skull but instead creates a mask by extending the brain surface outward by 3 mm. In contrast, our model learns directly from imaging data, enabling it to adapt to anatomical variation and perform more accurately in real-world clinical settings.

To mitigate variability in measured volumes of extracranial tissues caused by defacing artifacts, we introduced a brain mask–based cropping strategy to define a consistent region of interest across subjects. MRI scan anonymization procedures (Familiar et al., 2024), which are commonly used to protect patient privacy, can inadvertently remove or alter extracranial tissues, making it difficult to measure consistently. The standardized ROI maintained spatial consistency across datasets and proved reliable for skull and muscle estimation. It is notable that given the relatively small volume of the subcutaneous fat in the extracranial region, small variations in registration alignment can translate into large changes in absolute percentage.

We demonstrated how TissUnet-derived extracranial tissue volume offers a biologically meaningful context in modeling lipid profiles during youth. Monitoring cholesterol during adolescence provides valuable insight for identifying early cardiometabolic risk (Hyperlipidemia in Children | Symptoms, Diagnosis & Treatment, n.d.), yet integrating imaging with biochemical markers at scale has been limited by manual segmentation constraints. In the ABCD study, 888 participants had both T1-weighted MRI and blood lipid data available, enabling population-level analysis of extracranial fat volumes—an approach previously infeasible without labor-intensive expert annotation. While the temporalis muscle has emerged as a validated T1w-based surrogate marker for sarcopenia, existing methods are largely restricted to 2D cross-sectional area (CSA) or manual estimation (Hsieh et al., 2019; Zapaishchykova, Liu, et al., 2023). However, beyond 2D temporalis muscle segmentation, no established pipelines currently exist for systematic volumetric analysis of extracranial tissues—including skull, muscle, and fat—despite their potential to yield additional prognostic insights. Such automated MRI-derived measures may be particularly valuable in pediatric populations who routinely undergo neuroimaging, including childhood cancer survivors and those with chronic neurologic conditions. Future studies should evaluate whether volumetric temporalis muscle measurements outperform CSA in clinical risk prediction and functional outcomes.

This study has several limitations. First, T1-weighted fat-saturated sequences, although beneficial for suppressing fat signals that may obscure intracranial structures, render extracranial fat largely invisible, making it challenging to validate fat segmentation on such scans. Consequently, the generalizability of our model to fat-saturated images remains uncertain. Second, while the model was designed to tolerate defacing, segmentation performance can degrade under extreme conditions. For instance, in the UK Biobank dataset, extensive cropping of the temporalis muscle restricts accurate volume estimation. Future work should explore domain adaptation strategies to extend model applicability to neurodegenerative conditions, such as Alzheimer’s disease. Moreover, prospective clinical studies are needed to assess the downstream clinical relevance of automated extracranial tissue measurements.

## 5 Conclusion

We present TissUnet, a robust deep learning-based pipeline for segmenting extracranial structures—skull, muscle, and fat—in T1-weighted brain MRIs. Trained on pseudo-labels from CT data and validated across diverse pediatric and adult settings, including brain tumor datasets, TissUnet enables accurate tissue quantification and introduces automated skull thickness measurement. By addressing common challenges in extracranial segmentation, including defacing and limited annotation availability, our method supports comprehensive neuroimaging and anthropometric analyses for future applications in clinical research, growth assessment, and treatment planning.

## Supplementary Material

Supplementary Material

## Data Availability

The complete dataset (Supplementary Methods 1) aggregated for this study contains primary datasets that differ widely in terms of their “openness,” that is, their availability for secondary use without restrictions or special efforts by the team. Preliminary studies ranged from fully open and downloadable datasets in the public domain to more restricted datasets that could only be used for specific purposes, under separate agreements, or after special efforts had been made to provide data in shareable form. The model weights, training, and test code are available at https://github.com/AIM-KannLab/TissUNet.
