# Supplementary material for: TissUnet: Improved extracranial tissue and cranium segmentation for children through adulthood
Source: Imaging Neurosci (Camb). 2026 Jan 5;4:IMAG.a.1067. doi: 10.1162/IMAG.a.1067 (PMC12770910; doi:10.1162/IMAG.a.1067)
Supplement: Supplementary Material [file IMAG.a.1067_supp.pdf]

Supplementary Methods

Supplementary Methods 1. Datasets

CERMEP

CERMEP is multi-modal database of 37 healthy subjects constructed with MRI, CT and [^{18}F]FDG PET images. For all participants, the PET/CT scan and the MRI session took place on the same day (between 8 a.m. and 14 p.m.). PET and CT data were acquired on a Siemens Biograph mCT64. The subjects' MR and PET images were visually reviewed by two neurologists for conspicuous brain abnormalities. MRI sequences were obtained on a Siemens Sonata 1.5 T scanner. Three-dimensional anatomical T1-weighted sequences (MPRAGE) were acquired in sagittal orientation (TR 2400 ms, TE 3.55 ms, inversion time 1000 ms, flip angle 8°). The images were reconstructed into a 160 × 192 × 192 matrix with voxel dimensions of 1.2 × 1.2 × 1.2 mm3 (axial field of view 230.4 mm). Sagittal Fluid-Attenuated Inversion Recovery (FLAIR, [15]) images (TR 6000 ms, TE 354 ms, Inversion time 2200 ms, flip angle 180°) were acquired with a 176 × 196 × 256 matrix and a voxel size of 1.2 × 1.2 × 1.2 mm3 (Mérida et al., 2021).

ACRIN-TCIA

Adult patients newly diagnosed with pathologically confirmed GBM (World Health Organization [WHO] grade IV) that had visible residual disease after surgical resection, and planned for initial treatment with radiation therapy (RT) and temozolomide (TMZ), with or without additional agents, were enrolled. Amount of residual tumor did not impact eligibility and visible residual disease included T2/FLAIR hyperintensity. The study enrolled the first patient in March 2010 and the last in August 2013, with follow up ending 1 year later (July 2014). Of the 50 patients enrolled, 42 had evaluable imaging MR studies and 38 patients had evaluable ^{18}F-FMISO PET scans relating to the primary aim. Additionally, 37 patients had evaluable DSC imaging, 31 had evaluable DCE imaging, 39 had evaluable diffusion tensor imaging (DTI) data, 17 had evaluable spectroscopy (MRS) data and 13 patients had BOLD imaging that has never been analyzed. For each MR imaging session, patient scans were completed on 1.5 or 3 T scanners (Philips 3T (12 patients), GE 3T (12 patients), Siemens 3T (2 patients), and Siemens 1.5T (five patients) magnets). The current protocol can be found online (Protocol-ACRIN 6684 Amendment 7, 01.24.12) ("ACRIN-FMISO-BRAIN," n.d.).

BRATSPeds

The BraTS-PEDs dataset includes a retrospective multi-institutional cohort of conventional/structural magnetic resonance imaging (MRI) sequences, including pre- and post-gadolinium T1-weighted (labeled as T1 and T1CE), T2-weighted (T2), and T2-weighted fluid attenuated inversion recovery (T2-FLAIR) im- ages, from 464 pediatric high-grade glioma. Inclusion criteria comprised of pediatric subjects with: (1) histologically- approved high-grade glioma, i.e., high-grade astrocytoma and diffuse midline glioma (DMG), including radiologically or histologically-proven diffuse intrinsic pontine glioma (DIPG); (2) availability of all four structural mpMRI sequences on treatment-naive imaging sessions. Exclusion criteria consisted of: (1) images assessed to be of low quality or with artifacts that would not allow for reliable tumor segmentation; and (2) infants younger than one month of age. Data for 464 patients was obtained

through CBTN (n =120), DMG/ DIPG Registry (n = 256), Boston's Children Hospital (n = 61), and Yale University (n = 27) (Kazerooni et al., 2024)

SynthRad

This dataset consists of a total of 1080 CT and MRI/CBCT image pairs that were acquired between 2018 and 2022 in the radiation oncology departments of three Dutch university medical centers: University Medical Center Utrecht, University Medical Center Groningen, and Radboud University Medical Center. All patients in this dataset have been treated with external beam radiotherapy in the brain or pelvic region (photon or proton beam therapy). For anonymity, we will refer to the three centers with centers A, B, and C without specifying which letter belongs to which center. This dataset is presented as part of the SynthRAD2023 challenge (https://synthrad2023.grand-challenge.org/), which is structured into two tasks: task 1 addresses MR-to-CT image synthesis and hence consists of MR/CT image pairs, task 2 focuses on CBCT-to-CT image translation and consists of CBCT/CT image pairs. Two anatomical regions were considered for each task: the brain and the pelvis. Inclusion criteria were the treatment with radiotherapy and the acquisition of CT and either an MRI for treatment planning (task 1) or a CBCT for patient positioning during image-guided radiotherapy (task 2). Case selection in the brain was blind to clinical information concerning primary tumor etiology, making the tumor characteristics a random sample of the clinical routine. During data collection, no gender restrictions were considered, and the dataset consists of 64% male subjects and 36% female subjects. A mostly adult patient population was collected, with patients aged 3 to 93 years and a mean age of 65. For task 1, MRIs were acquired with a T1-weighted gradient echo or an inversion prepared—turbo field echo (TFE) sequence and collected along with the corresponding planning CTs for all subjects. The collected MRIs of centers B and C were acquired with a Gadolinium contrast agent, while the MRIs selected from center A were acquired without contrast (Table 2). No contrast was acquired for CT (Thummerer et al., 2023).

ABCD

Data used in the preparation of this article were obtained from the Adolescent Brain Cognitive Development SM (ABCD) Study (https://abcdstudy.org), held in the NIMH Data Archive (NDA). This is a multisite, longitudinal study designed to recruit more than 10,000 children age 9-10 and follow them over 10 years into early adulthood. The ABCD Study® is supported by the National Institutes of Health and additional federal partners under award numbers U01DA041048, U01DA050989, U01DA051016, U01DA041022, U01DA051018, U01DA051037, U01DA050987, U01DA041174, U01DA041106, U01DA041117, U01DA041028, U01DA041134, U01DA050988, U01DA051039, U01DA041156, U01DA041025, U01DA041120, U01DA051038, U01DA041148, U01DA041093, U01DA041089, U24DA041123, U24DA041147. A full list of supporters is available at abcdstudy.org [https://abcdstudy.org/federal-partners.html]. A listing of participating sites and a complete listing of the study investigators can be found at abcdstudy.org/consortium_members[https://abcdstudy.org/consortium_members/].
ABCD consortium investigators designed and implemented the study and/or provided data but did not necessarily participate in the analysis or writing of this report. This manuscript reflects the authors' views and may not reflect the opinions or views of the

NIH or ABCD consortium investigators. The ABCD data repository grows and changes over time. The ABCD data used in this report came from the fast-track data release. The raw data are available at NDA [https://nda.nih.gov/edit_collection.html?id=2573]). Additional support for this work was made possible from supplements to U24DA041123 and U24DA041147, the National Science Foundation (NSF 2028680), and Children and Screens: Institute of Digital Media and Child Development Inc (Casey et al., 2018a).

PING
This dataset was created by the NIH to support research on typical brain and behavioral development. It includes clinical, behavioral, and neuroimaging data collected from a large sample of children and adolescents between the ages of 4 and 18, recruited from multiple research sites across the United States. Participants aged 6 to 17 provided written assent to take part in the study. MRI scans were acquired using either General Electric or Siemens 1.5 Tesla scanners (Rivkin et al., 2010).

Calgary
The Preschool MRI study in The Developmental Neuroimaging Lab at the University of Calgary uses different magnetic resonance imaging (MRI) techniques to study brain structure and function in early childhood (OSF [https://osf.io/axz5r/files/osfstorage]). All imaging for this dataset was conducted using the same General Electric 3T MR750w system and 32-channel head coil (GE, Waukesha, WI) at the Alberta Children's Hospital in Calgary, Canada. Children were scanned either while awake and watching a movie, or while sleeping without sedation. The University of Calgary Conjoint Health Research Ethics Board (CHREB) approved this study (REB13-0020). T1-weighted images were acquired using an FSPGR BRAVO sequence with TR = 8.23 ms, TE = 3.76 ms, TI = 540 ms, flip angle=12 degrees, voxel size = 0.9x0.9x0.9 mm3, 210 slices, matrix size=512x512, field of view=23.0 cm. ASL images were acquired with the vendor supplied pseudo continuous 3D ASL sequence with TR = 4.56 s, TE = 10.7 ms, in-plane reso- lution of 3.5x3.5 mm2, post label delay of 1.5 s, and thirty 4.0 mm thick slices. The sequence scan time was 4.4 minutes (Reynolds et al., 2020b)

BabyConnectome
The Baby Connectome Project (BCP [https://nda.nih.gov/edit_collection.html?id=2848]) is a four-year study of children from birth through five years of age, intended to provide a better understanding of how the brain develops from infancy through early childhood and the factors that contribute to healthy brain development. This project is a research initiative of the Neuroscience Blueprint – a cooperative effort among the 15 NIH Institutes, Centers, and Offices that support neuroscience research. The BCP is supported by Wyeth Nutrition through a donation to the FNIH. Images are acquired on 3T Siemens Prisma MRI scanners using a Siemens 32-channel head coil at the Center for Magnetic Resonance Research (CMRR) at the University of Minnesota and the Biomedical Research Imaging Center (BRIC) at the University of North Carolina at Chapel Hill (Howell et al., 2019)

ICBM
Data used in the preparation of this work were obtained from the International

Consortium for Brain Mapping (ICBM) database (ICBM [www.loni.usc.edu/ICBM]). The ICBM project (Principal Investigator John Mazziotta, M.D., University of California, Los Angeles) is supported by the National Institute of Biomedical Imaging and BioEngineering. ICBM is the result of efforts of co-investigators from UCLA, Montreal Neurologic Institute, University of Texas at San Antonio, and the Institute of Medicine, Juelich/Heinrich Heine University - Germany. Data collection and sharing for this project was provided by the International Consortium for Brain Mapping (ICBM; Principal Investigator: John Mazziotta, MD, PhD). The National provided ICBM funding Institute of Biomedical Imaging and BioEngineering. ICBM data are disseminated by the Laboratory of Neuro Imaging at the University of Southern California (Mazziotta et al., 2001)

IXI

The data [https://brain-development.org/ixi-dataset/] has been collected at three different hospitals in London: Hammersmith Hospital using a Philips 3T system (details of scanner parameters [http://brain-development.org/scanner-philips-medical-systems-intera-3t/]), Guy's Hospital using a Philips 1.5T system (details of scanner parameters [http://brain-development.org/scanner-philips-medical-systems-gyroscan-intera-1-5t/]), and Institute of Psychiatry using a GE 1.5T system (details of the scan parameters not available at the moment). The Thames Valley MREC granted ethical approval. The T1 and T2 images were acquired prior to diffusion-weighted imaging using 3D MPRAGE and dual-echo weighted imaging.(*The IXI Dataset*, n.d.)
